# The Usefulness of Over‐the‐Scope Clips for Managing Gastrointestinal Complications of Endoscopic or Surgical Procedures: The SAGA‐OTSC Registry Study

**DOI:** 10.1002/jgh3.70316

**Published:** 2025-12-20

**Authors:** Daisuke Yamaguchi, Takashi Akutagawa, Takahiro Yukimoto, Naoyuki Tominaga, Koichi Miyahara, Hiroharu Kawakubo, Ryuma Morita, Norihiro Okamoto, Yutaro Fujimura, Kento Sadashima, Hironobu Takedomi, Takuya Shimamura, Nanae Tsuruoka, Yasuhisa Sakata, Ryo Shimoda, Motohiro Esaki

**Affiliations:** ^1^ Division of Gastroenterology, Department of Internal Medicine, Faculty of Medicine Saga University Saga Japan; ^2^ Department of Endoscopic Diagnostics and Therapeutics Saga University Hospital Saga Japan; ^3^ Department of Gastroenterology National Hospital Organization Ureshino Medical Center Ureshino Japan; ^4^ Department of Gastroenterology Saga‐Ken Medical Centre Koseikan Saga Japan; ^5^ Department of Internal Medicine Karatsu Red Cross Hospital Saga Japan; ^6^ Department of Gastroenterology Imari Arita Kyouritsu Hospital Saga Japan

**Keywords:** bleeding, complications, fistula, over‐the‐scope clip, perforation

## Abstract

**Background:**

The over‐the‐scope clip (OTSC) system enables full‐thickness closure of gastrointestinal wall defects and has been used to address gastrointestinal complications of endoscopic and surgical procedures such as perforation, bleeding, and fistulae. This study aimed to evaluate the use of the OTSC system in managing these complications in Saga Prefecture, Japan.

**Methods:**

We retrospectively analyzed the clinical data of 23 patients who underwent OTSC system‐based management for complications of surgical or endoscopic procedures between January 2020 and July 2024 across five institutions. All procedures were performed by expert endoscopists.

**Results:**

The mean patient age was 72.3 years and 10 were men. Indications for OTSC treatment included perforation (52.2%), fistula (21.7%), and bleeding (13.0%). Fifteen cases (65.2%) involved the upper gastrointestinal tract. The median duration from the onset of complication to OTSC application was 4 days. OTSC treatment was technically successful in 21 patients (91.3%). Mean procedure time was 39.7 min. Treatment failure occurred in two cases involving jejunal lesions. Seven patients including two technical failures (30.5%) required additional treatment (surgery or repeat OTSC). For patients who required additional intervention, the median duration from OTSC to further treatment was 21 days. The timing of OTSC treatment was not associated with treatment success or the need for further intervention.

**Conclusions:**

The OTSC system demonstrated a high success rate and favorable safety profile for managing gastrointestinal tract complications. However, early surgical intervention may be required in case OTSC treatment is applied for lesions of deep small bowel or large fibrotic defects.

**Abbreviations**
ALT: alanine aminotransferase
APTT: activated partial thromboplastin time
AST: aspartate aminotransferase
BMI: body mass index
BUN: blood urea nitrogen
CONSORT: Consolidated Standards of Reporting Trials
CRP: C‐reactive protein
IQR: interquartile range
LDH: lactate dehydrogenase
OTSC: over‐the‐scope clip
PT: prothrombin time

## 1 Introduction

Recent advances in endoscopic procedures for gastrointestinal diseases have significantly expanded therapeutic options, allowing for organ preservation while achieving outcomes comparable to those of surgery. However, even when careful precautions are taken, complications such as perforation, bleeding, fistula formation, and anastomotic leakage can occur with both types of intervention. Effective strategies for preventing and managing these complications are essential to ensure patient safety and successful outcomes [1, 2, 3, 4, 5, 6, 7, 8].

The over‐the‐scope clip (OTSC) system (Ovesco Endoscopy GmbH, Tübingen, Germany) is a soft endoscopic, full‐thickness suturing device designed to close defects in the gastrointestinal tract. Initially introduced by Kirschniak et al. in 2007, it has a straightforward mechanism that provides strong tissue approximation [9]. The OTSC exerts secure closure and has shown clinical utility in managing refractory gastrointestinal conditions such as bleeding, perforation, fistulae, and anastomotic leakage, and has gained widespread use in clinical practice [10, 11, 12, 13, 14, 15, 16, 17, 18, 19, 20, 21, 22, 23, 24]. In addition, specialized grasping forceps facilitate the closure of relatively large defects or perforations [10].

In the present study, we collected the experiences of OTSC treatment from our affiliated hospitals to investigate clinical outcomes of surgical or endoscopic complications managed by the procedure, aiming to improve endoscopic practices for complication management at regional core hospitals.

## 2 Methods

### 2.1 Study Design and Data Collection

This retrospective observational study was conducted in five hospitals in Saga Prefecture, Japan. Eligible patients were identified through a comprehensive review of electronic medical records. Data entry was performed by the principal investigator, a designated physician at each participating institution, or by research collaborators under their supervision.

**FIGURE 1:**
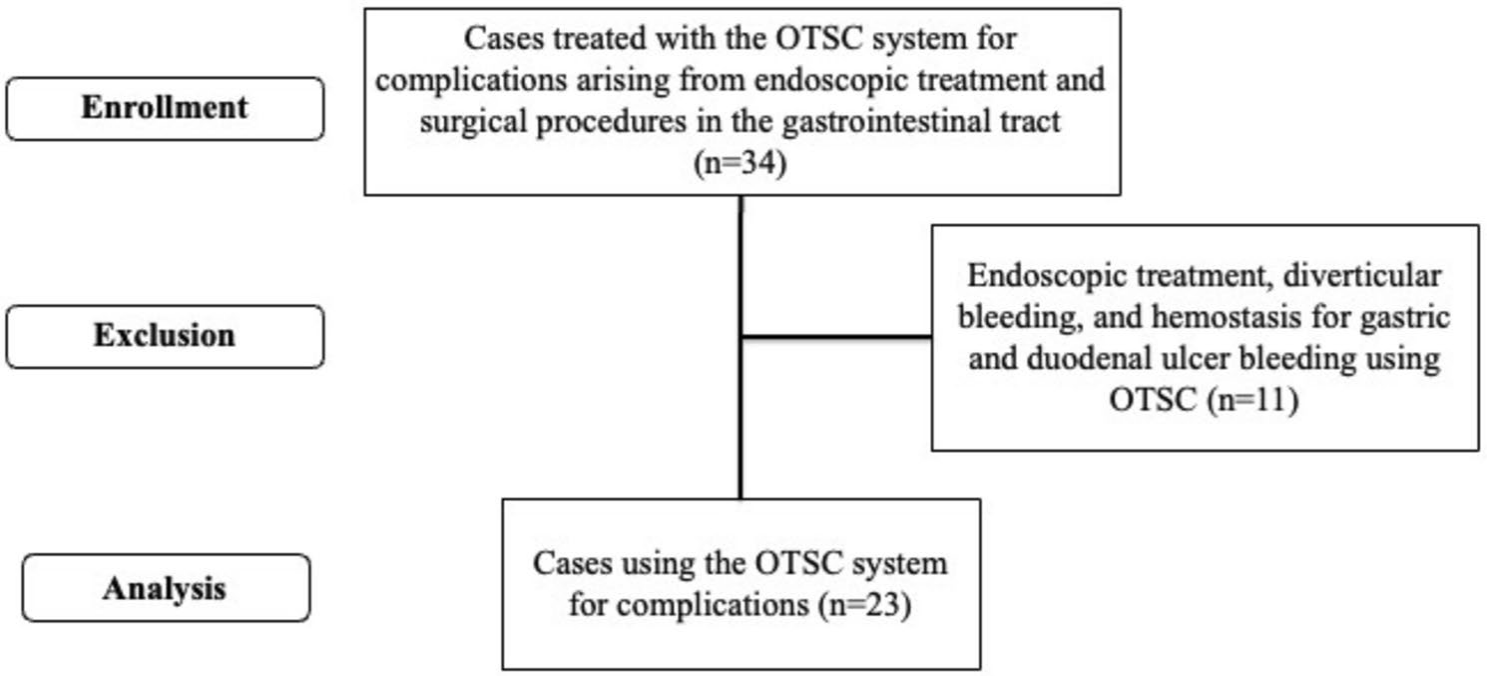
Study flowchart.

Thirty‐four patients who underwent OTSC system‐based treatment between January 2012 and July 2024 were identified and reviewed. Eleven were excluded because the OTSC system was used for indications other than complication management [25]. The 23 patients in whom the system was used specifically for managing complications of surgical or endoscopic procedures were included for analysis (Figure 1).

Demographic, clinical, diagnostic, procedural (both primary and OTSC procedures), and laboratory data were extracted from the electronic medical record and recorded. Details regarding the primary disease, the development of procedure‐related complications, the interval between complication onset and OTSC‐based intervention, and outcomes and complications of OTSC‐based treatment were also documented.

The study was conducted in accordance with the tenets of the Declaration of Helsinki and is reported using the guidelines of the Consolidated Standards of Reporting Trials. The study protocol and the consent procedure were approved by the Saga University Clinical Research Review Board (approval number 2024‐05‐03, approved on August 5, 2024).

### 2.2 OTSC Procedure

The OTSC system comprises an applicator cap preloaded with a shape‐memory nitinol alloy clip, a hand wheel, and a thread retriever. The deployment mechanism is straightforward: by turning the hand wheel, which is connected to the endoscope's working channel, the thread pulls the mounted clip forward and releases it onto the targeted site. The clip, which features four prongs, anchors the tissue from the left and right sides, enabling consistent compression of the lesion. Because of the open space between the prongs, blood flow is preserved, preventing tissue necrosis and promoting wound healing [9].

For lesions that cannot be adequately drawn into the applicator cap by suction alone, auxiliary grasping devices such as the twin grasper and anchor forceps (Ovesco Endoscopy) may be used. The twin grasper forceps can alternately open and close two independent jaws, making it suitable for approximating the edges of large defects. In contrast, the anchor forceps deploy three retractable needles simultaneously, offering a better grip in firm or fibrotic tissue.

Different clip designs are available depending on the clinical indication: a gastrostomy closure type with long and thick prongs for gastric closure, a traumatic type with short and sharp prongs for use in thin‐walled organs such as the small and large intestines, and an atraumatic type intended for hemostasis in bleeding lesions. We mainly used a therapeutic endoscope with a working channel diameter ≥ 3.2 mm and a water‐jet endoscope (GIF‐Q260J or GIF‐290T; Olympus, Tokyo, Japan). However, in cases where auxiliary forceps were needed, a double‐channel therapeutic scope (e.g., GIF‐2T240, Olympus) with a 3.7‐mm working channel was used [10, 11].

The procedure was carried out by expert endoscopists with prior experience using the OTSC system. The steps were as follows: (i) The hand wheel was attached to the endoscope's working channel, and the applicator cap with the mounted clip was affixed to the distal tip of the endoscope using the thread retriever. (ii) The optimal positioning for OTSC deployment was determined upon reaching the target site. (iii) Simple suction was employed if the entire defect could be aspirated into the cap. The twin grasper or anchor forceps were used when this was not feasible based on lesion characteristics. (iv) Once proper positioning and traction were achieved, the hand wheel was turned swiftly, similar to the technique used in endoscopic variceal ligation, and the clip was released to complete tissue closure. In cases where a single clip was insufficient to close the entire defect, additional clips were applied as necessary [10].

### 2.3 Statistical Analysis

Continuous variables with a normal distribution are expressed as means with standard deviation and were compared using the Student's t‐test. Continuous variables with a non‐normal distribution are expressed as medians with interquartile range (IQR) and were compared using the Wilcoxon rank‐sum test. Categorical variables are presented as frequencies with percentage and were compared using the chi‐squared test. *p* < 0.05 was considered significant. Statistical analyses were performed using JMP 17.0.0 (SAS Institute Inc., Cary, NC, USA).

## 3 Results

### 3.1 Patient Demographics and Clinical Background

Among the 23 patients who underwent OTSC treatment for gastrointestinal complications and were included for analysis, the mean age was 73.3 ± 12.0 years. Ten patients (43.5%) were men. The mean body mass index was 21.0 ± 2.6 kg/m^2^. Over half of the patients (56.5%) had a history of abdominal surgery. Alcohol consumption was reported in seven patients (30.4%) and four (17.4%) were current or former smokers. Antithrombotic therapy was prescribed in seven patients (30.4%). The most common comorbidities were hypertension (60.9%), malignant tumor (26.1%), and cardiovascular disease (21.7%). Patient characteristics are shown in detail in Table 1.

**TABLE 1:** Patient characteristics.

Patient's characteristics	
Number of patients (*N*)	23
Age (years)	73.3 ± 12.0
Sex, male	10 (43.5%)
BMI (kg/m^2^)	21.0 ± 2.6
History of abdominal surgery	13 (56.5%)
Alcohol drinking	7 (30.4%)
Smoking	4 (17.4%)
Antithrombotic medication	7 (30.4%)
*Comorbidity*	
Cardiovascular diseases	5 (21.7%)
Cerebrovascular diseases	4 (17.4%)
Chronic kidney diseases	2 (8.7%)
Chronic liver damage	2 (8.7%)
Diabetes mellitus	2 (8.7%)
Dyslipidemia	2 (8.7%)
Hypertension	14 (60.9%)
Malignant tumor	6 (26.1%)

*Note:* Data shown are number of patients (%) or mean ± standard deviation.

Abbreviation: BMI, body mass index.

### 3.2 Indications for OTSC and Initial Laboratory Findings

As shown in Table 2, the primary indications for OTSC use in the 23 patients were as follows: perforation (12 patients, 52.2%), fistula (5 patients, 21.7%), bleeding (3 patients, 13.0%), and incomplete suturing (3 patients, 13.0%). Initial laboratory data did not reveal severe abnormalities (Table 2). There were no signs of severe anemia (mean hemoglobin, 11.1 ± 2.2 g/dL), marked inflammatory response (mean CRP, 3.3 ± 5.6 mg/dL), or significant hepatic or renal dysfunction (mean AST, 36.2 ± 31.9 U/L; mean ALT, 36.1 ± 29.5 U/L; mean creatinine, 0.8 ± 0.4 mg/dL). These results indicate that most patients did not present with advanced systemic deterioration at the time of OTSC application.

**TABLE 2:** Indications for over‐the‐scope clip system use and initial laboratory data.

Cause of disease using OTSC	
Perforation	12 (52.2%)
Fistula	5 (21.7%)
Bleeding	3 (13.0%)
Incomplete suture	3 (13.0%)

Initial laboratory data	
White blood cell (k/μL)	7.4 ± 4.2
Hemoglobin (g/dL)	11.1 ± 2.2
Platelets (k/μL)	25.1 ± 13.1
PT‐INR	1.07 ± 0.2
Total Protein (g/dL)	6.2 ± 1.1
Albumin (g/dL)	3.2 ± 1.0
Serum AST (U/L)	36.2 ± 31.9
Serum ALT (U/L)	36.1 ± 29.5
Serum total bilirubin (mg/dL)	0.8 ± 1.0
Serum BUN (mg/dL)	15.7 ± 7.4
Serum creatinine (mg/dL)	0.8 ± 0.4
CRP (mg/dL)	3.3 ± 5.6

*Note:* Data shown are number of patients (%) or mean ± standard deviation.

Abbreviations: ALT, alanine aminotransferase; APTT, activated partial thromboplastin time; AST, aspartate aminotransferase; BUN, blood urea nitrogen; CRP, C‐reactive protein; OTSC, over‐the‐scope clip; PT‐INR, international normalized ratio of prothrombin time.

### 3.3 Procedural Outcomes and Follow‐Up

The OTSC procedure was successfully completed in 21 patients (91.3%). In the two unsuccessful cases, the lesions involved the jejunum, where complete closure could not be achieved. The mean procedure time was 39.7 ± 42.3 min. The OTSC was applied at various anatomical sites as follows: esophagus (six patients, 26.1%), stomach (six patients, 26.1%), duodenum (three patients, 13.0%), jejunum (two patients, 8.7%), and colon (six patients, 26.1%). The median duration from the onset of complication to OTSC application was 4 [IQR, 1–4] days. For patients who required additional intervention after OTSC, the median duration from OTSC to further treatment was 21 [IQR, 18–51] days. Following OTSC application, 16 patients (69.3%) were managed conservatively, four (17.4%) underwent surgery, and three (13.0%) underwent repeat OTSC treatment. Adverse events were rare: two patients developed hypotension (8.6%) and one experienced cardiopulmonary arrest (4.3%). The latter patient was immediately resuscitated successfully. OTSC treatment outcomes are shown in detail in Table 3.

**TABLE 3:** Outcomes of over‐the‐scope clip system treatment.

Treatment outcome	
Successful endoscopic procedure	21 (91.3%)
Procedure time (min)	39.7 ± 42.3
*Location of OTSC*	
Esophagus	6 (26.1%)
Stomach	6 (26.1%)
Duodenum	3 (13.0%)
Jejunum	2 (8.7%)
Colon	6 (26.1%)
*Operator of OTSC*	
Specialists	23 (100%)
Number of days until OTSC treatment after complications (days)	4 [1–4]
Number of days until additional treatment after OTSC	21 [18–51]
*Follow‐up after OTSC*	
Observation treatment	16 (69.6%)
Surgery	4 (17.4%)
Re‐OTCS	3 (13.0%)
Adverse event	
Hypotension	2 (8.6%)
Cardiopulmonary arrest Rescue	1 (4.3%)

*Note:* Data shown are number of patients (%), mean ± standard deviation, or median [interquartile range].

Abbreviation: OTSC, over‐the‐scope clip.

### 3.4 Representative Case: Successful Endoscopic Closure of Esophageal Perforation

**FIGURE 2:**
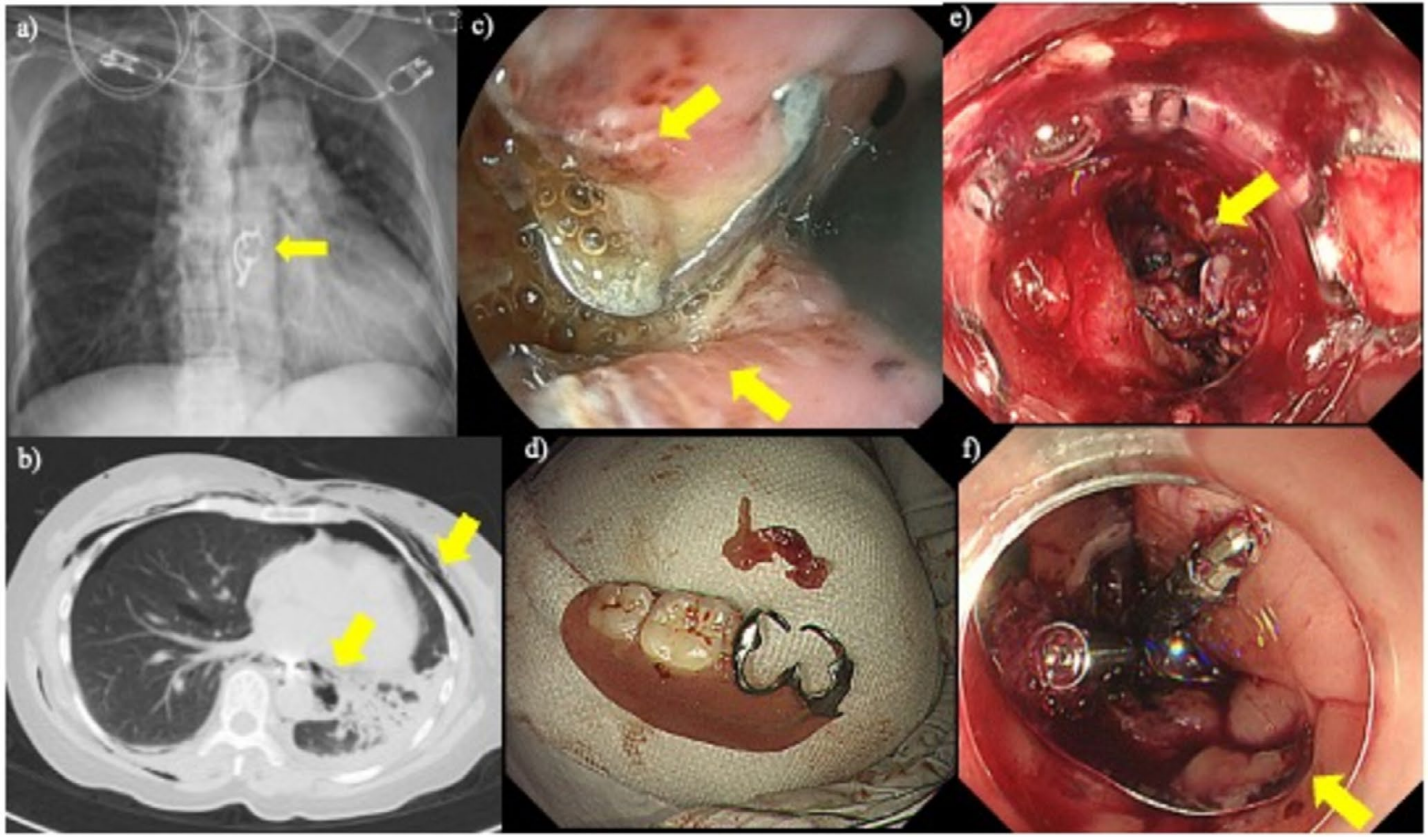
Endoscopic removal of esophageal foreign body (denture). (a) Esophageal foreign body (denture) on plain chest radiography (arrow). (b) Free air in the mediastinum on chest computed tomography (arrow). (c) Perforation of the middle esophagus caused by the foreign body (arrow). (d) Foreign body (denture) after removal. (e) The over‐the‐scope clip system was attached and perforation confirmed (arrow). (f) The endoscopic closure was successful (arrow).

A 61‐year‐old woman with no significant medical history was referred to our hospital after discovering that her denture was missing following a meal. Plain radiography and computed tomography of the chest revealed mediastinal emphysema and located the denture in the thoracic esophagus (Figure 2a,b). Endoscopy revealed a large perforation of the mid‐esophagus (Figure 2c).

Under general anesthesia, a double‐scope technique was employed using a therapeutic scope (GIF‐290T; Olympus) and a transnasal endoscope (EG‐L580NW7; Fujifilm, Tokyo, Japan). The two endoscopes grasped each end of the denture and maneuvered it into the stomach for safe transoral retrieval (Figure 2d).

**FIGURE 3:**
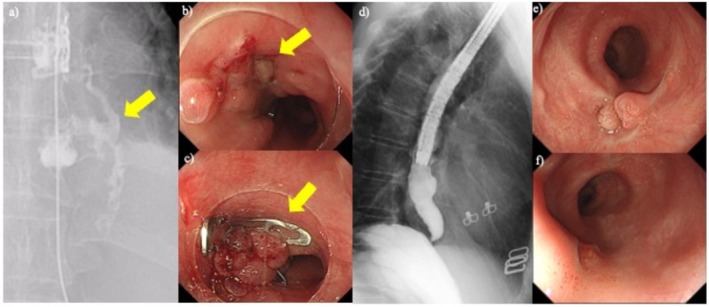
Evaluation after closure and treatment of persistent fistula. (a) Esophagus barium swallow test 17 days after over‐the‐scope clip (OTSC) closure showed that the fistula remained. (b) Endoscopy showed the fistula on the anal side. (c) OTSC closure was performed again. (d) Repeat evaluation 77 days later showed no contrast agent leakage outside the lumen. (e, f) Complete closure was confirmed by endoscopy.

A large esophageal defect was subsequently closed using the OTSC system and reinforced with Mantis clips (Boston Scientific, Marlborough, MA, USA) (Figure 2e,f). Seventeen days after the initial procedure, endoscopy revealed a residual fistula on the anal side of the initial closure (Figure 3a,b), for which an additional OTSC was placed (Figure 3c). Contrast imaging 77 days after repeat OTSC application demonstrated no extraluminal leakage, and endoscopy confirmed complete healing of the perforation site (Figure 3d–f).

This case illustrates the effectiveness of OTSC application for treating large esophageal perforations, a condition that often requires invasive surgery such as esophagectomy. In this case, successful endoscopic management obviated the need for surgery, demonstrating the clinical significance and therapeutic potential of the OTSC system in managing severe iatrogenic gastrointestinal complications.

### 3.5 Failure and Re‐Intervention Outcomes

Details of the two technical failures and the five cases that required subsequent therapy are summarized in Table 4. Both technical failures involved jejunal lesions and both patients were being treated for postoperative defects in the jejunum (one iatrogenic perforation during endoscopic retrograde cholangiopancreatography and one jejuno‐cutaneous fistula). Despite the use of a traumatic‐type clip and ancillary grasping forceps, complete closure could not be achieved owing to marked fibrosis. Both patients ultimately underwent elective fistula closure surgery 18 and 21 days, respectively, after the OTSC procedure.

**TABLE 4:** Summary of technical failures and cases that required re‐intervention.

Failure	Age	Gender	Initial OTSC site	Treatment time (min)	Primary diagnosis	Reason for OTSC use	Comorbidity	Additional treatment days after OTSC (days)	Re‐treatment details	Duration of hospitalization (days)
Case1	74	Female	Jejunum	22	Common bile duct stones	Postoperative jejunal perforation during ERCP	Gastric cancer surgery, Bronchial asthma	21	Fistula closure surgery	74
Case2	79	Female	Jejunum	49	Duodenal papillary cancer	Postoperative jejuno‐cutaneous fistula	Cerebral infarction, Hypertension	18	Fistula closure surgery	293
*Surgery*										
Case3	53	Female	Sigmoid colon	19	Ovarian cancer	Pelvic abscess with sigmoid colon‐vaginal fistula	Breast cancer	18	Stoma creation surgery	77
Case4	68	Male	Gastroduodenal anastomosis	15	Early gastric cancer	Postoperative anastomotic fistula	Hepatic cirrhosis	137	Fistula excision surgery	156
*Re‐OTSC*										
Case5	61	Female	Esophagus	75	Foreign body ingestion (denture)	Esophageal perforation due to foreign body ingestion	None	17	Repeated OTSC for fistula	47
Case6	59	Male	Esophagus	27	Idiopathic esophageal rupture	Fistula after idiopathic esophageal rupture	Sequela of cerebral hemorrhage, Hypertension	68	Repeated OTSC for fistula	132
Case7	72	Male	Esophagogastric anastomosis	15	Esophageal cancer	Postoperative anastomotic fistula	Hypertension, Bronchial asthma	28	Repeated OTSC for fistula	165

Abbreviation: OTSC, over‐the‐scope clip.

Surgical conversion after successful OTSC was required in two patients. Surgery was required for one sigmoid colon–vaginal fistula and one gastroduodenal anastomotic leak 18 and 137 days after the procedure, respectively. Complete healing was eventually achieved in both patients.

Repeat OTSC was required in three patients: two with a mid‐esophageal perforation/fistulae and one with an anastomotic fistula at the esophagogastric junction. A second clip was applied between 17 and 68 days after the first procedure. Endoscopic salvage was successful in all three patients.

Overall, the reintervention‐free rate was 69.6%, and no procedure‐related mortality occurred. There was no significant association between the timing of the first OTSC application (≤ 4 vs. > 4 days) and the composite outcome of technical failure or reintervention (*p* = 0.41).

## 4 Discussion

This multicenter retrospective study demonstrated that the OTSC system is a safe and effective treatment modality for managing gastrointestinal complications such as perforation, fistula, and anastomotic failure, even in regional core hospitals in Japan. Technical success of OTSC treatment was achieved in 91.3% of procedures, and most patients recovered without surgical intervention, underscoring the clinical value of OTSC in real‐world practice.

The representative case of a large foreign body‐induced esophageal perforation described above illustrates the therapeutic potential of the OTSC system. Although such injuries have traditionally required radical surgery such as esophagectomy, OTSC treatment with concurrent use of Mantis clips achieved complete endoscopic closure, resulting in uneventful healing. This case highlights both the strength of the full‐thickness tissue grasp and the safety profile of the device in high‐risk scenarios.

Several features explain the favorable outcomes obtained with OTSC: the delivery system is user‐friendly; the clip confers strong tissue apposition and full‐thickness capture; and the open‐prong design preserves microvascular perfusion, thereby preventing ischemic necrosis and promoting mucosal healing [10, 21]. These advantages have expanded the indications for its use, ranging from first‐line hemostasis of non‐variceal bleeding [12, 13, 14, 15, 22, 23, 24] to closure of iatrogenic perforations, postoperative leaks, and chronic fistulae [11, 12, 13, 16, 17, 18, 19, 20].

Nevertheless, there are clinical scenarios in which OTSC treatment alone may be insufficient, as shown in Table 4. All technical failures occurred in the jejunum similar to previous reports, suggesting deep small‐bowel sites to be challenging for the procedure: the wall is thin, scope maneuverability is limited, and postoperative defects tend to be large and fibrotic [21]. Based on our experience, defects larger than 20 mm in diameter and/or marked fibrosis in the jejunum warrant early surgical consultation and consideration of prompt operative management.

Conversely, repeat OTSC application again demonstrated highly effective for persistent upper GI fistulae, since the procedure achieved durable closure in two esophageal lesions and esophagogastric anastomosis. Esophageal lesions are difficult to close with OTSC due to the narrow space and the direction of the lesion. However, as has been consistent with results for laparoscopic sleeve gastrectomy [19], we could demonstrate the usefulness of repeat OTSC for the esophageal or esophagogastric anastomotic complications. It thus seems beneficial to assess and attempt repeat OTSC after insufficient closure to avoid surgery.

Despite initial clip success, 17% of patients ultimately required subsequent surgery, emphasizing that OTSC should be viewed as one component of a multimodal algorithm rather than a stand‐alone solution. Importantly, all surgically managed patients recovered without major morbidity, which supports the safety of a sequential endoscopic‐then‐surgical management when warranted.

Additional practical considerations include device cost (approximately 80 000 JPY per clip), the learning curve associated with safe deployment, and technical difficulties posed by indurated or fibrotic lesions. Although expert endoscopists performed every procedure in this series, structured training programs will be necessary to generalize the OTSC treatment for nonexpert endoscopists.

This study has inherent limitations: its retrospective design, without a control group, introduces selection bias; the modest sample size and incomplete long‐term follow‐up may underestimate the incidence of late complications or recurrences. Future prospective multicenter registries are therefore needed to confirm the predictive factors for OTSC failure identified here (jejunal location, lesion size > 20 mm, and marked fibrosis) and to optimize the timing of reintervention.

In conclusion, the OTSC system is highly effective for the vast majority of gastrointestinal defects and bleeding events encountered in core hospitals. However, there seems to be anatomical (deep small bowel) and lesion‐specific (large, fibrotic defects) contexts in which early surgical intervention should be necessary.

## Funding

The authors have nothing to report.

## Consent

The authors have nothing to report.

## Conflicts of Interest

The authors declare no conflicts of interest.

## Data Availability

The data that support the findings of this study are available from the corresponding author upon reasonable request.
